# Dealing with daily challenges in dementia (deal-id study): effectiveness of the experience sampling method intervention ’Partner in Sight’ for spousal caregivers of people with dementia: design of a randomized controlled trial

**DOI:** 10.1186/s12888-016-0834-5

**Published:** 2016-05-11

**Authors:** Rosalia J. M. van Knippenberg, Marjolein E. de Vugt, Rudolf W. Ponds, Inez Myin-Germeys, Frans R. J. Verhey

**Affiliations:** Department of Psychiatry and Neuropsychology and Alzheimer Centre Limburg, School for Mental Health and Neurosciences, Maastricht University, Maastricht, The Netherlands; Department of Neurosciences, Center for Contextual Psychiatry, KU Leuven, Leuven, Belgium

**Keywords:** Dementia, Caregiver, Support, ESM intervention, RCT

## Abstract

**Background:**

There is an urgent need for psychosocial interventions that effectively support dementia caregivers in daily life. The Experience Sampling Methodology (ESM) offers the possibility to provide a more dynamic view of caregiver functioning. ESM-derived feedback may help to redirect caregivers’ behavior towards situations that elicit positive emotions and to increase their feelings of competence in the caretaking process. This paper presents the design of a study that evaluates the process characteristics and effects of the ESM-based intervention ‘Partner in Sight’.

**Methods/design:**

A randomized controlled trial with 90 spousal caregivers of people with dementia will be conducted. Participants will be randomly assigned to the experimental (6-week ESM intervention including feedback), pseudo-experimental (6-week ESM intervention without feedback), or control group (care as usual). Assessments will be performed pre- and post-intervention and at 2-, and 6-month follow-up. Main outcomes will be sense of competence, perceived control, momentary positive affect, and psychological complaints (depressive symptoms, perceived stress, anxiety, momentary negative affect). In addition to the effect evaluation, a process and economic evaluation will be conducted to investigate the credibility and generalizability of the intervention, and its cost-effectiveness.

**Discussion:**

The potential effects of the ESM intervention may help caregivers to endure their care responsibilities and prevent them from becoming overburdened. This is the first ESM intervention for caregivers of people with dementia. The results of this study, therefore, provide a valuable contribution to the growing knowledge on m-health interventions for dementia caregivers.

**Trial registration:**

Dutch Trial Register NTR4847; date registered Oct 9, 2014.

**Electronic supplementary material:**

The online version of this article (doi:10.1186/s12888-016-0834-5) contains supplementary material, which is available to authorized users.

## Background

Caregivers of people with dementia (PwD) are at great risk of becoming overburdened and of developing psychological and physical symptoms during the caretaking process [1]. This calls for psychosocial interventions that effectively support caregivers of PwD in daily life and help them handling their care responsibilities.

Various psycho-social interventions have been developed in recent years for caregivers of PwD, including psycho-education, emotional support, practical assistance, cognitive-behavioral therapy, and multi-component interventions [2]. Overall, research has shown significant but small effects of current interventions on caregiver outcomes. A common feature in these studies is that outcome measures include retrospective self-assessments that are highly susceptible to emotional and cognitive biases [3]. Moreover, retrospective methods do not provide information about fluctuations in subjective experiences over time and across situations that caregivers of PwD may face due to the continually changing care demands.

The Experience Sampling Methodology (ESM) is an innovative approach in assessing subjective experiences in real-time within the flow of daily life. ESM consists of a structured diary method in which repeated self-assessments are electronically recorded the moment they occur, in their natural setting [4, 5]. ESM offers the possibility to provide a more accurate and detailed view of caregiver functioning, since it enables daily fluctuations in subjective experiences to be explored and it minimizes retrospective recall biases [6, 7]. Therefore, ESM might be a valuable addition to standard retrospective methods, particularly in older populations with an increased incidence of memory deficits [8].

Recently, there has been growing interest in adapting ESM to clinical practice. By using modern technology, such as personal digital assistants (PDAs) and apps, momentary data are immediately available to both caregivers and professionals. This creates the opportunity to develop ESM interventions that provide explicit, visualized feedback on implicit dynamic patterns of feelings, experiences, and behavior [9, 10]. Receiving feedback on behavior can result in emotional and behavioral change, something already known from the field of behavioral therapy [11, 12]. The feedback may help caregivers redirect their behavior towards situations that are conducive to positive emotional experiences. In this way, ESM offers the opportunity to actively involve caregivers in their own empowering process and to provide more personally tailored support [13]. Both these aspects have been demonstrated to be essential in effective psychosocial interventions [14, 15].

A focus on positive experiences facilitates a more positive interaction between the caregiver and the PwD and increases positive emotions in both the parties [16]. According to the ‘broaden-and-build theory’, positive emotions elevate the ability to cope with stressful situations and might consequently help to increase caregivers’ feelings of being capable of caring for the PwD [17, 18]. Positive emotions could thus be an important target in caregiver support interventions, increasing caregiver well-being and reducing long-term negative impacts, such as stress and burden [19, 20].

In a recent study, ESM-derived feedback on positive affect was provided to persons with depression during a 6-week intervention period. Its results showed that personalized feedback increased self-awareness and resulted in a significant decline in depressive symptoms [9]. In another ESM study, in which depressed individuals collected ESM data for scientific purposes but without receiving feedback, some participants reported that responding to the ESM questionnaires had already ‘helped them’ and enhanced their awareness of their daily functioning [21]. So far, few studies have applied ESM in caregivers of PwD in the context of research [22, 23]. Recent evidence suggests that ESM is a feasible method for use with this often elderly and vulnerable population (van Knippenberg et al.: Dealing with daily challenges in dementia (Deal-id study): an innovative approach to assess caregiver functioning in the flow of daily life, submitted). However, to date, no ESM interventions have been developed to support caregivers of PwD in dealing with daily challenges associated with dementia. This paper describes the design of a randomized controlled trial (RCT) to evaluate the effects of the ESM-based intervention program ‘Partner in Sight’ for spousal caregivers of PwD.

### Study aims

The specific objectives of the current study are:

1. *Process evaluation* to investigate the internal and external validity of the intervention based on sampling quality (recruitment, randomization, and reach) and intervention quality (relevance, feasibility, and performance according to protocol). The process evaluation will be conducted prior to the effect evaluation in order to provide essential information about credibility and generalizability [24].
2. *Effect evaluation* to assess whether ‘Partner in Sight’ is superior to a pseudo-intervention and control group in terms of producing a clinically significant increase in subjective well-being, as proven by an increase in caregivers’ sense of competence, perceived control, momentary positive affect, and a decrease in psychological complaints (depression, anxiety, stress, and momentary negative affect). A follow-up evaluation will be conducted to examine whether the effects have lasted two and six months after the intervention.
3. *Economic evaluation* to assess the cost-effectiveness of ‘Partner in Sight’ by estimating the impact of the intervention on resource use, costs, and health outcomes.

## Methods and design

**Fig. 1:**
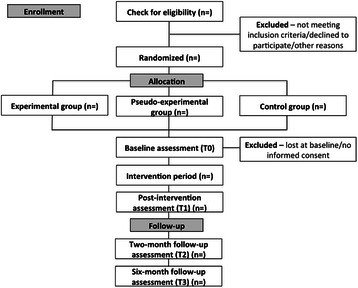
CONSORT flow diagram

The study is a randomized controlled trial with three treatment arms. The experimental condition in which caregivers participate in the ESM-intervention ‘Partner in Sight’ (ESM data collection including feedback) will be compared with a pseudo-experimental condition (ESM data collection without feedback) and a control group (care as usual). Data will be collected pre- and post-intervention and at two- and six-months follow-up (Fig. 1).

### Study population

The study population will consist of spousal caregivers of community dwelling people with all subtypes and stages of dementia. No age limit will be applied. Participants will be recruited in memory clinics (Maastricht University Medical Center + (MUMC+), Atrium Medical Center Parkstad), ambulatory mental health care institutions (Virenze-RIAGG Maastricht, Lionarons GGZ), dementia day care centers (Sevagram, NOVIzorg, Orbis Glana, Proteion, care farm Ransdalerveld), and caregiver support services (Hulp bij Dementie, Steunpunt Mantelzorg) in the southern region of the Netherlands, and via the website of the Dutch Alzheimer Society. The clinician or care counselor who is involved in the treatment of the PwD will approach caregivers to participate in the study. Subsequently, potential participants will be contacted and screened by the researcher to make sure that they fully meet the following inclusion criteria: (1) being a spousal caregiver of a person with a diagnosis of dementia; (2) sharing a household with the PwD; and (3) informed consent obtained. Exclusion criteria will be: insufficient cognitive abilities to engage in ESM; being overburdened or having severe health problems based on clinical judgment of a knowledgeable practitioner; taking care for a PwD caused by Human Immunodeficiency Virus (HIV), acquired brain injury, Down syndrome, chorea related to Huntington’s disease or alcohol abuse.

### Randomization

Caregivers will be randomly assigned to the experimental group, pseudo-experimental group, or control group. Randomization will be computer-generated and conducted by an independent statistician. Block randomization will be performed to diminish the risk of an unbalanced assignment to the three treatment arms. Randomly permuted blocks with variable block sizes (3, 6, and 9) will be used, by which the block size and specific order will be chosen randomly at the beginning of each block. This reduces the risk of predicting group assignment and keeps research staff blind to the randomization process. The design of this study is single-blind. An independent research assistant, who is being blinded to the treatment allocation, will conduct the baseline, post-intervention and follow-up assessments, and will be asked to evaluate success of blinding and reasons for possible unmasking on the Case Record Form.

### ESM procedure

ESM will be carried out using the PsyMate, an electronic touchscreen device that is specifically designed to monitor experiences and behavior in daily life and that offers the possibility to provide immediate ESM-derived feedback (www.psymate.eu). The PsyMate has been extensively studied and refined in several studies concerning psychiatric populations (e.g. psychosis and depression) [10, 13]. In a recent ESM study with spousal caregivers of PwD, the PsyMate was considered to be a user-friendly and easily accessible device (van Knippenberg et al.: Dealing with daily challenges in dementia (Deal-id study): an innovative approach to assess caregiver functioning in the flow of daily life, submitted).

The PsyMate will be programmed to generate ten beeps (sound and vibration) per day at random intervals between 7:30 AM and 10:30 PM. ESM will be used as an assessment tool during the baseline assessment (three consecutive days = 30 beeps in total) and the post assessment (three consecutive days = 30 beeps in total) to evaluate the effectiveness of the intervention. Furthermore, ESM will be used as an intervention tool during the 6-week intervention period (three consecutive days per week = 10×3×6 = 180 beeps in total). In order to include different days of the week, the PsyMate will beep alternately on Friday, Saturday, and Sunday, or Tuesday, Wednesday, and Thursday during the intervention period.

After each beep, caregivers will be asked to complete a questionnaire presented on the screen of the PsyMate, including current affect (four positive affect and eight negative affect items), self-esteem (four items), physical well-being (four items), as well as current context and activities (daily life activities, social company, location and events). At the end of each questionnaire, caregivers will have to indicate whether the beep disturbed them. Answering all questions will take approximately three minutes per beep (van Knippenberg et al.: Dealing with daily challenges in dementia (Deal-id study): an innovative approach to assess caregiver functioning in the flow of daily life, submitted). Additionally, the PsyMate will be programmed to generate a morning and evening questionnaire at the beginning and end of each day. The morning questionnaire consists of six items regarding their sleep quality during the previous night and their current level of energy. The evening questionnaire contains twenty-five items concerning the caregivers’ daily sense of well-being and competence, and neuropsychiatric symptoms in the PwD during that day. Responses will be rated on 7-point Likert scales (ranging from 1 ‘not at all’ to 7 ‘very much’), bipolar scales (ranging from −3 ‘very unpleasant/very unimportant’ to +3 ‘very pleasant/very important’), a Visual Analogue Scale (ranging from 0 ‘worst imaginable health’ to 100 ‘best imaginable health’), and box-checking formats. Responses cannot be corrected afterwards. An overview of the ESM items with corresponding response choices and concepts is presented in the Appendix. The specific items of the ESM questionnaires were developed and selected according to information available from previous ESM studies [25, 26], knowledge about the experiences and situations that caregivers of PwD could be expected to encounter in daily life, and the guidelines for item development created by ESM experts [27]. Moreover, the ESM questionnaires were recently tested in a feasibility study that yielded positive results (van Knippenberg et al.: Dealing with daily challenges in dementia (Deal-id study): an innovative approach to assess caregiver functioning in the flow of daily life, submitted).

### Study procedure

#### Baseline assessment (T0)

After the randomization procedure, a baseline assessment (T0) will take place in the caregiver’s home or at the MUMC+ according to their preference. Participants will be asked by the research assistant to sign the informed consent before continuation of the study procedure. Next, a demographical interview will be conducted to assess caregiver and care recipient characteristics. Additionally, caregivers will be asked to participate in a 3-day ESM baseline measurement, starting the day after the baseline assessment. A 30-min briefing will be provided during the baseline assessment to ensure that they fully understand the procedure and how to operate the PsyMate. A demo questionnaire will be presented to familiarize caregivers with the device and a leaflet containing all relevant information regarding the use of the device will be handed out. Finally, the caregivers will be asked to complete a number of retrospective questionnaires at their own convenience as part of the effect evaluation.

#### Intervention period

##### Experimental group

Caregivers in the experimental group will participate in the 6-week intervention ‘Partner in Sight’ and collect ESM data for three consecutive days a week. Every two weeks they will receive ESM-derived feedback in a face-to-face session with a coach in their home or at the MUMC+ according to their preference. This sums up to a total of three feedback sessions. The aim of the feedback sessions is to provide an overview of the caregiver’s everyday functioning, including their mood (i.e. levels of positive affect), daily life activities, and social interactions. The focus will be on positive rather than negative emotional experiences and how these relate to specific daily contexts. In this way, we try stimulate caregivers to redirect their behavior towards situations that elicit positive emotions.

At the beginning of each feedback session average levels of positive affect experienced during the past two weeks will be presented. Subsequently, a feedback module on *daily activities* and/or *social interactions in daily life* will be discussed with the caregiver. Feedback modules are based on an existing ESM intervention, which has proven to be effective in people with depression [9]. During the first feedback session the module ‘*daily activities’* will be discussed with the caregiver. The data may, for example, illustrate that positive emotions increase during moments of active relaxation, while the caregiver is actually spending the least amount of time on active relaxation activities. The coach will stimulate caregivers to think about the findings and to implement new insights into their daily lives. In the second feedback session the module ‘*social interactions in daily life’* will be added. A caregiver might state, for example, that he prefers to spend his time alone, without any company. The feedback, however, may show that positive emotions are experienced particularly while being in company instead of when being alone. This finding might induce an increase in social interactions, which in turn may lead to increased positive emotions. The third session will combine both modules. At the end of each session the weekly progress in levels of positive affect yielded by daily activities and/or social interactions will be evaluated. A more detailed description of the feedback modules is presented in Table 1.

**Table 1:** Feedback modules of the intervention ‘Partner in Sight’

Session	Feedback Module	Description
1	1 Daily activities	- Pie chart of the average time spent on different activities (e.g. caring for partner, household, active relaxation, passive relaxation, resting, self care) during the past two weeks
		- Graph demonstrating the relationship between different kind of activities and experienced levels of positive affect
		- Pie chart of the average time spent on active relaxation in the presence and absence of the PwD
		- Graph showing experienced levels of positive affect during active relaxation in the presence and absence of the PwD
		- Pie chart of the average time spent on passive relaxation in the presence and absence of the PwD
		- Graph showing experienced levels of positive affect during passive relaxation in the presence and absence of the PwD
2	1 & 2 Social interactions	Added:
		- Pie chart of the average time spent in different kinds of company (e.g. partner, friends, family, colleagues, alone) during the past two weeks
		- Graph demonstrating the relationship between different types of social company and experienced levels of positive affect
3	1 & 2	See description above
1,2,3	General graphs	- Graph including information on average levels of positive affect during the past two weeks
		- Graph demonstrating the weekly progress in levels of positive affect during the course of the intervention
		- Graph demonstrating the weekly progress in levels of positive affect yielded by daily activities
		- Graph demonstrating the weekly progress in levels of positive affect yielded by social interactions

*PwD* person with dementia

The coach will present all feedback verbally and graphically (in clear pie charts and bar graphs) to the caregiver according to a standardized protocol. Before the start of the study all coaches will receive training with clear instructions on how to provide feedback. After each session both the caregiver as well as the involved clinician or care counselor will be provided with a written copy of the feedback.

At the end of each feedback session there will be a debriefing concerning the use of the PsyMate, e.g. difficulties operating the device, technical problems, items that are unclear, and reasons for missing measurements. Furthermore, the participant will be reminded and encouraged to fill out the PsyMate during the following two weeks as accurately as possible conform the instructions given by the coach. During the third feedback session the coach will additionally conduct an unstructured interview to assess the feasibility of the intervention.

##### Pseudo-experimental group

A pseudo-experimental group is added to the study design to investigate whether the ESM-derived feedback adds value to the use of the PsyMate without receiving any direct feedback. Repeated self-assessments might already increase caregivers’ self-awareness and redirect their behavior accordingly [21].

Caregivers in the pseudo-experimental group will participate in a 6-week pseudo-intervention and collect ESM data for three days a week. Every two weeks they will receive a face-to-face session with a coach. However, during these sessions they will not be provided with feedback on their daily recordings. Alternatively, a semi-structured interview on their well-being during the past two weeks will be performed to prevent any effects of different duration of contact with the coach.

At the end of each session caregivers will, similar to the participants in the experimental group, be provided with a debriefing concerning the use of the ESM-device.

##### Control group

Caregivers in the control group will receive care as usual during the 6-week intervention period. Care conditions will differ among caregivers and will be registered carefully at baseline. In general, care as usual includes low-frequent sessions with a clinician from the memory clinic or counselor from a caregiver support service.

#### Post-intervention assessment (T1)

After the intervention period a post-intervention assessment will be executed in the caregiver’s home or at the MUMC+ according to their preference. As part of the post-intervention assessment, caregivers will be asked to participate in a 3-day ESM post measurement. They will be shortly briefed with respect to the procedure. Afterwards, all participants will be asked to complete a questionnaire concerning their general experiences with the ESM-device during the complete study period. Based on their answers a semi-structured interview will be conducted to discuss the questionnaire. Finally, caregivers will be asked again to complete a number of retrospective questionnaires at their own convenience as part of the effect evaluation.

#### Follow-up assessments (T2 & T3)

A two- and six-month follow-up assessment will be administered in which participants receive a number of retrospective questionnaires by post and are asked to return them after completion.

### Retention

Participants will be provided with periodic newsletters to inform them about the current status of the study, plans for the next phase, as well as to acknowledge their support.

### Instruments

For an overview of the instruments used during the baseline, post-intervention and follow-up assessments see Table 2.

**Table 2:** Flowchart of measures used during the assessments

	Pre-test	Intervention period			Post-tests		
	T = 0	FB1	FB2	FB3	T = 1	T = 2	T = 3
Primary outcome measures RCT							
- Sense of competence: SSCQ	X				X	X	X
- Perceived control: PMS	X				X	X	X
Secondary outcome measures RCT							
- Depressive symptoms: CES-D	X				X	X	X
- Perceived stress: PSS	X				X	X	X
- Anxiety symptoms: HADS-A	X				X	X	X
ESM outcome measures RCT							
- Momentary positive affect	X				X		
- Momentary negative affect	X				X		
Additional measures RCT							
- Demographic variables	X						
- Neuropsychiatric symptoms in PwD: NPI-Q	X				X	X	X
- Quality of the relationship: 4 items of the University of Southern California Longitudinal Study of Three-Generation Families measures of positive affect	X				X	X	X
- Coping: UCL	X				X	X	X
- Personality: subscale neuroticism of NEO-FFI	X				X	X	X
Outcome measures process evaluation							
- Subjective experiences with intervention: quantitative questionnaire & qualitative semi-structured interview				X			
- Subjective experiences with the use of the ESM device: quantitative questionnaire					X		
- Subjective experiences with ESM procedure: quantitative questionnaire		X	X	X	X		
Outcome measures economic evaluation							
- Resource use: RUD-lite	X				X	X	X
- Quality of life: EQ-5D	X				X	X	X

*RCT* randomized controlled trial, *SSCQ* Short Sense of Competence Questionnaire, *PMS* Pearlin Mastery Scale, *CES-D* Center for Epidemiological Studies Depression Scale, *PSS* Perceived Stress Scale, *HADS-A* Hospital Anxiety and Depression Scale, Anxiety subscale, *PwD* person with dementia, *NPI-Q* Neuropsychiatric Inventory Questionnaire, *UCL* Utrechtse Coping List, *NEO-FFI* NEO Five-Factor Inventory, *ESM* Experience Sampling Methodology, *RUD-lite* Resource Utilization in Dementia – shortened version, *EQ-5D* EuroQoL-5D

#### Primary outcome measures

*Sense of Competence*: caregivers’ subjective feelings of competence will be assessed with the Short Sense of Competence Questionnaire (SSCQ), a shortened version of the 27-item Sense of Competence Questionnaire (SSQ) [28]. The SSCQ consists of seven items, rated on a 5-point scale from 1 (agree very strongly) to 5 (disagree very strongly). The items reflect three domains of caregivers’ feelings of being capable to care for the PwD: satisfaction with their own performance as a caregiver (2 items), satisfaction with the PwD as a care recipient (3 items), and consequences of involvement in care for personal life of the caregiver (2 items). A total sum score (range 7–35) will be calculated for each participant. Higher sum scores represent higher levels of sense of competence. The scale displays good content and construct validity in previous research [28].

*Perceived control:* the extent to which a person perceives him- or herself to be in control of events and on-going situations, also known as mastery, will be measured with the Pearlin Mastery Scale (PMS) [29]. The scale contains seven items with scores varying from 0 (completely agree) to 4 (completely disagree). Items are summed to form a total mastery score (range 0–28), with higher scores indicating greater perceived control. The psychometric properties of the PMS are good according to previous research [30].

#### Secondary outcome measures

*Depressive symptoms:* the Center for Epidemiological Studies Depression Scale (CES-D) will be used to assess depressive symptoms among caregivers [31]. It includes twenty items that rate the frequency of symptoms during the past week. Item scores range from 0 (rarely or none of the time present [less than 1 day]) to 3 (most or all of the time present [5–7 days]). The total sum score ranges from 0 to 60, with higher scores indicating more depressive symptoms. Items depict major components of depressive symptomatology, such as depressed mood, feelings of guilt and worthlessness, feelings of helplessness and hopelessness, psychomotor retardation, loss of appetite, and sleep disturbance. The CES-D has been widely used in research on caregiving and has proven to be sensitive to changes in caregiver depressive symptoms after intervention [14].

*Perceived stress:* the Perceived Stress Scale (PSS) will be used to measure the degree to which situations in one’s life are appraised as stressful [32]. The PSS consists of ten items, rated on a 5-point scale from 0 (never) to 4 (very often), regarding unpredictability, control, and overload. Total sum scores on the PSS range from 0 to 40, with higher scores representing higher levels of stress. Adequate validity and reliability has been demonstrated in previous research [32].

*Anxiety symptoms:* the 7-item anxiety subscale of the Hospital Anxiety and Depression Scale (HADS) will be employed to assess the severity of anxiety symptoms in caregivers [33]. Item scores range from 0 (not at all) to 3 (a great deal of the time) and will be accumulated to produce a total sum score (range 0–21), with higher scores indicating more anxiety. The HADS has frequently been used in caregivers of PwD and has shown good reliability rates [34].

#### ESM outcome measures

*Momentary positive and negative affect:* the ESM data collected during the 3-day ESM baseline measurement and 3-day ESM post measurement will be used to assess caregivers’ momentary positive and negative affect. Positive affect will be defined as the mean score of the items: ‘I feel cheerful’, ‘I feel relaxed’, ‘I feel enthusiastic’, and ‘I feel satisfied’. Negative affect will be defined as the mean score of the items ‘I feel insecure’, ‘I feel lonely’, ‘I feel anxious’, ‘I feel irritated’, ‘I feel down’, ‘I feel desperate’, ‘I feel tensed’, and ‘I feel confident’. A mean positive and negative affect score will be calculated for each completed beep during the day, with higher scores indicating higher levels of positive and negative affect.

#### Additional measures

*Demographics:* demographic variables, including age, sex, and level of education of both the caregiver and the care recipient, will be assessed during a demographical interview with the caregiver. Furthermore, information about the type, severity, and duration of dementia, caregiver hours of contact with the PwD, caregiver hours of caring for the PwD, and PwD hours spent in a dementia day care setting will be collected. The Clinical Dementia Rating scale (CDR) will be used to stage the severity of the dementia [35]. The CDR score is rated on a 5-point scale: 0 = ‘normal’; 0.5 = ‘very mild dementia’; 1 = ‘mild dementia’; 2 = ‘moderate dementia’; and 3 = ‘severe dementia’. The CDR has become widely accepted in the clinical setting as a reliable and valid global assessment measure of dementia [36].

*Neuropsychiatric symptoms in the PwD:* the Neuropsychiatric Inventory Questionnaire (NPI-Q), a brief form of the Neuropsychiatric Inventory (NPI), will be used to evaluate neuropsychiatric symptoms in the PwD and associated caregiver distress [37]. The NPI-Q evaluates twelve neuropsychiatric domains, including: delusions, hallucinations, agitation/aggression, dysphoria/depression, anxiety, euphoria, apathy, disinhibition, irritability, aberrant motor behavior, nighttime behavioral disturbances, and appetite and eating abnormalities. For each domain, the caregiver answers a screening question to indicate whether the symptom is present or not. If present, the severity is rated on a scale from 1 (mild) to 3 (severe). Total sum scores range from 0 to 36. Additionally, a caregiver distress score is rated for each domain on a 6-point scale, ranging from 0 (not emotionally stressful) to 5 (extremely stressful). The Dutch version of the NPI-Q has been investigated and appears to be a valid instrument [38].

*Quality of the relationship:* quality of the relationship will be assessed using four items of the University of Southern California Longitudinal Study of Three-Generation Families measures of positive affect [39]. The items represent: general closeness, communication, similarity of views on life, and degree of getting along with each other. Answer scales range from 1 (not at all) to 4 (very). Caregivers will answer these items in terms of the current situation and to what extent the relationship changed since illness onset (1 = much better, 5 = much worse). Summed scores will be used as an index of the change in relationship quality. A previous study found good internal reliability [40].

*Coping:* the 44-item Utrechtse Coping List (UCL) will be used to measure seven different coping strategies in the caregiver, including seeking distraction (eight items), expressing emotions (three items), seeking social support (six items), avoiding (eight items), fostering reassuring thoughts (five items), passive coping (seven items), and active coping (seven items). Items are rated on a 4-point scale, ranging from 1 (rarely or never use this strategy) to 4 (very often use this strategy). The reliability and validity has been found sufficient despite some inconsistencies in the literature [41].

*Personality:* the 12-item Neuroticism domain of the NEO Five-Factor Inventory (NEO-FFI) will be used to identify subjects who are susceptible to psychological distress. This domain measures six traits: anxiety, angry hostility, depression, self-consciousness, impulsiveness, and vulnerability. Item scores will be rated on a 5-point scale, ranging from 0 (strongly disagree) to 4 (strongly agree), and will be accumulated to generate a total sum score (range 0–48). The reliability and internal consistency of the Dutch version of the NEO-FFI is good [42].

#### Outcome measures in the process evaluation

As part of the process evaluation, sampling quality and intervention quality will be evaluated to determine the internal and external validity of the intervention program and to reveal facilitators and barriers for the intervention.

*Sampling quality*: will be evaluated by describing (1) the recruitment and randomization procedure; (2) the informed consent and allocation procedure; and (3) barriers and facilitators to the recruitment of caregivers. Reach will be established by the proportion of caregivers participating and the number of institutions involved in the intervention.

*Intervention quality:* will be evaluated by determining (1) the relevance of the intervention; (2) the feasibility of the intervention; and (3) the extent to which the intervention was performed according to protocol.

Required data will be collected from the research database, and during the sessions with the coach and the post-intervention assessment. Objective measures of compliance with respect to the ESM procedure (number of completed beeps) and the intervention (number of drop-outs) will be examined. Technical problems with the PsyMate will be logged. Subjective experiences with respect to the intervention (i.e. clearness, relevance, usability, impact on daily functioning, satisfaction with procedure and time burden, suggestions for improvement, and recommendations to other caregivers) will be assessed by means of a quantitative questionnaire and a qualitative interview with the caregiver during the third feedback session. In addition, subjective experiences with the PsyMate and ESM procedure (e.g. difficulties operating the device, clearness items, readability of the items on the screen, interference with daily activities, and reasons for missed beeps) will be examined.

Performance of the intervention according to protocol will be evaluated with a structured registration form, which includes protocol deviations and duration of each face-to-face session during the intervention period.

#### Economic evaluation

The economic evaluation will involve a cost-effectiveness analysis (CEA) and includes the assessment of costs as well as outcomes of the intervention.

*Resource use and costs:* the Resource Utilization in Dementia – shortened version (RUD-lite) will be used to map the utilization of resources for both the PwD and the caregiver [43]. The RUD-lite assesses both formal and informal resource use, making it possible to calculate costs from a societal point of view. Costs will be calculated by multiplying the quantity of resource use by the cost price per resource unit and will include the period from the baseline assessment until the last follow-up assessment (6 months). All relevant costs will be determined according to the Dutch guidelines for cost calculations in health care [44].

*Intervention costs:* intervention costs will include the amount of time spent on briefing the caregivers regarding the PsyMate and ESM procedure, the feedback sessions with the coach, administration (e.g. writing short feedback reports), telephone contact, the training session for the coach on how to provide the feedback, and costs of required materials (e.g. PsyMate). The coach will register the duration of the feedback sessions, administration, and telephone contact with the caregiver on a structured registration form. Incremental cost effectiveness ratios (ICER) will be calculated by dividing the difference in total costs between the treatment arms by the difference in average effect size.

*Quality of life:* caregivers’ health related quality of life will be measured with the EuroQol-5D (EQ-5D) and consists of five items representing the following dimensions mobility, self-care, usual activities, pain/discomfort, and anxiety/depression [45]. Respondents indicate their health state by rating each dimension on a 3-level scale, including 1 ‘no problems’, 2 ‘moderate problems’, or 3 ‘severe problems’. In addition, the EQ-VAS will be used to record caregivers’ self-rated health status on a Visual Analogue Scale, anchored at ‘the best health you can imagine’ (100) and ‘the worst health you can imagine’ (0). The EuroQol demonstrates a good test-retest reliability [46]. For the cost-effectiveness analysis, quality-adjusted life-years (QALYs) will be calculated according to the Dutch EuroQol tariff [47].

### Sample size

The calculation of the sample size will be based on previous studies using the Sense of Competence Questionnaire (SCQ) as outcome measure in intervention studies for caregivers of PwD, the use of repeated measures, within-between interaction with a mean effect size of 0.8 [48], and the following assumptions: alpha 0.05, 80 % power and 25 % loss to follow up. Accordingly, we aim to enrol 90 participants in the study (30 participants per group).

### Statistical analyses

#### Primary and secondary outcome measures

Data will be entered twice to guarantee data integrity. Before the analyses, data will be checked for missing values and handled according to their distribution (missing completely at random, missing at random, or not missing at random), and the ‘intention-to-treat’ principle. Additionally, data will be checked for outliers, and normality. Non-parametric tests will be used in case of non-normality after data transformation. Possible baseline differences in group characteristics will be tested with t-tests for continuous and *χ*^2^-tests for categorical variables.

To investigate changes in the primary and secondary outcomes for each group during the intervention period, an analysis of covariance (ANCOVA) will be performed with outcome at post-intervention as the dependent variable, group (experimental, pseudo-experimental, control) as the between-subject factor, and outcome at baseline and potential confounders (e.g. demographic variables, coping, personality, and quality of the relationship) as covariates. Separate analyses will be conducted for each outcome variable. Group differences in the post-intervention outcome, adjusted for the baseline value, will be examined to test the effectiveness of the intervention. In case group differences are present, the inter-group effect size will be calculated using Cohen’s *d*.

To examine changes in the primary and secondary outcomes for each group during the total study period, a linear mixed model (LMM) for repeated measures will be conducted. Analyses will be performed with group as a fixed between-subject factor (3 levels: experimental, pseudo-experimental, and control group) and time as fixed within-subject variable (4 levels: baseline, post-intervention, two-month follow-up, and six-month follow-up) and first-order interactions as additional fixed factors. The LMM will estimate fixed effects (regression slopes) for change in the intervals during (T0-T1) and after (T1-T2, T2-T3) the intervention period. The intervals will be entered as a categorical dummy variable (3 levels). Potential confounders will be added to the model as covariates. Additionally, coach will be added as a random factor to estimate the variability ascribed to the coach.

All data will be analysed using STATA 12.1 [49]. Tests of significance will report mean change and will be two tailed with *α* set at 0.05.

#### ESM outcome measures

Subjects have to complete at least 33 % of the ESM beeps in order to be included in the ESM data analysis [4]. To investigate changes in momentary positive and negative affect between the 3-day ESM baseline and post measurement for each group, an LMM will be performed for both outcome measures. LMMs are ideally suited for ESM data, since these models take into account the hierarchical data structure in which multiple ESM observations (beep level 1) are nested within days (day level 2), and days are nested within subjects (subject level 3). The XTMIXED command in STATA 12.1 [49] will be used to conduct analyses with group (3 levels: experimental, pseudo-experimental, and control group), time (2 levels: baseline and post-intervention), and the two-way interaction between group and time as fixed factors. Random slopes, representing positive or negative affect, will be added at the subject, day, and beep level. Potential confounders will be included in the model as covariates.

#### Process evaluation

Descriptive analyses will be conducted to summarize response rates, overall experiences regarding the ESM intervention and the PsyMate, and additional findings with respect to the sampling and intervention quality.

### Confidentiality

All study-related information will be stored securely at the study site. All participant information will be identified by a coded ID number to maintain participant confidentiality. The principal investigators and project team members will be able to access the data. All records that contain names or other personal identifiers (e.g. informed consent forms) will be stored separately from study records identified by code number. All datasets will be password protected.

### Monitoring and participant safety

The study will be monitored externally by the trial monitoring committee of the MUMC+ (Clinical Trial Center Maastricht). The trial monitoring committee is independent of the study organizers. All adverse events (AEs) and serious adverse events (SAEs) that occur during the study will be recorded during the (feedback) sessions, the post-intervention assessment, and the two- and six-month follow-up assessment. SAEs will be reported to the accredited Medical Ethical Committee that approved the protocol.

### Withdrawal of participants

Participants can leave the study at any time without justification, and without any consequences.

## Discussion

The current paper presents the study protocol of an RCT to evaluate the effectiveness of an ESM intervention to improve caregivers’ sense of competence and control, and psychological well-being. The potential effects of the intervention may help caregivers to keep on caring and prevent them from developing (more) health problems and becoming overburdened. This study contains several unique aspects. To our knowledge, this is the first ESM intervention for caregivers of PwD. ESM might be a promising tool in both research and clinical practice, since it offers the possibility to provide more detailed information on caregiver functioning in the flow of daily life. In this study we will use ESM as an intervention tool, but also as an assessment tool to evaluate the effects of the intervention. Therefore, an important advantage is that the results of this study represent a high level of ecological validity. Another unique aspect is that our intervention focuses on positive rather than negative experiences during the caretaking process. Positive emotions are important facilitators of adaptive coping and might increase caregivers’ feelings of being capable to care [18, 50]. The ESM intervention might enhance caregivers’ self-awareness and redirect their behavior to situations that are conducive to positive emotional experiences. Moreover, the intervention includes ESM-derived feedback that is tailored to the caregivers’ personal situation and actively involves them in their own empowering process. Both are known to be important aspects of effective caregiver support interventions [14]. The inclusion of a pseudo-experimental condition in our study design allows us to unravel the added value of the ESM-derived feedback to the use of the PsyMate in general. Caregivers might benefit from the use of the PsyMate without receiving any feedback, since repeated self-assessments may lead people to pay more attention to their internal states and own behavior [21]. Lastly, the intervention program will be delivered in daily practice and offers the possibility to be integrated in future clinical practice. The additional process and economic evaluation will provide valuable information on potential facilitators and barriers to implementation, and on the cost-effectiveness of the intervention.

Certain limitations need to be acknowledged in advance. There is a possibility of sample bias. Although participants will be recruited in a wide range of different institutions, the group that agrees to participate in the study might differ from the group that refuses to participate. In general, participants are expected to be relatively young, more highly educated, and more pro-active in seeking for support [51]. Moreover, recent results from our feasibility study, in which caregiver were asked to collect ESM data for only six consecutive days, showed that a large number of caregivers refused to participate because of a too busy time schedule (van Knippenberg et al.: Dealing with daily challenges in dementia (Deal-id study): an innovative approach to assess caregiver functioning in the flow of daily life, submitted). The even more time-intensive nature of the current study might, therefore, cause a selection bias towards caregivers that are not yet facing extreme difficulties in the caregiving process. It is also expected that a number of participants will drop out of the ESM intervention prematurely. In this specific population, it is likely that participants drop out for specific disease related reasons, such as institutionalization or death of the PwD. In a previous ESM study with persons with depression, 15 % of the participants did not complete the 6-week ESM intervention period [9]. The face-to-face sessions with a coach and the delivery of personalized feedback, however, might increase caregivers’ motivation to participate given that usual care for dementia caregivers often does not, or very infrequent, include counseling [52].

In conclusion, the results of this study will provide a valuable contribution to the growing knowledge on m-health interventions for caregivers of PwD. Our ESM intervention ‘Partner in Sight’ is expected to be effective in terms of caregiver well-being and might be an innovative approach to support caregivers of PwD in managing daily challenges during the course of the disease.

### Ethics approval and consent to participate

The Medical Ethical Committee of the MUMC+ approved this study (#143040). All participants gave informed consent to participate in the study.

#### Protocol amendments

Important protocol modifications, which may impact on the conduct of the study or may affect participant safety, including changes in study objectives, study design, study population, sample sizes, study procedures, or significant administrative aspects will require a formal amendment to protocol. Substantial amendments will be notified to the principal investigators and approved by the Medical Ethical Committee of the MUMC+ prior to implementation. Minor changes to the protocol that have no effect on the way the study is conducted will not be notified to the Medical Ethical Committee of the MUMC+, but will be recorded and filed by the investigators.

### Consent for publication

Not applicable.

### Availability of data

Not applicable.

### Availability of supporting data

Not applicable.

### SPIRIT Guidelines

This study protocol is based on the SPIRIT 2013 Statement [53]. A SPIRIT checklist is provided as an additional file.

## Appendix

**Table 3:** Description of the ESM concepts, items and response choices in the daily, morning and evening questionnaire

Daily questionnaire		
Concept	Item	Rating scale
Positive affect	1. I feel cheerful	7-point scale (1 ‘not at all’ to 7 ‘very much’)
	2. I feel relaxed	7-point scale (1 ‘not at all’ to 7 ‘very much’)
	3. I feel enthusiastic	7-point scale (1 ‘not at all’ to 7 ‘very much’)
	4. I feel satisfied	7-point scale (1 ‘not at all’ to 7 ‘very much’)
Negative affect	5. I feel insecure	7-point scale (1 ‘not at all’ to 7 ‘very much’)
	6. I feel lonely	7-point scale (1 ‘not at all’ to 7 ‘very much’)
	7. I feel anxious	7-point scale (1 ‘not at all’ to 7 ‘very much’)
	8. I feel irritated	7-point scale (1 ‘not at all’ to 7 ‘very much’)
	9. I feel down	7-point scale (1 ‘not at all’ to 7 ‘very much’)
	10. I feel desperate	7-point scale (1 ‘not at all’ to 7 ‘very much’)
	11. I feel confident	7-point scale (1 ‘not at all’ to 7 ‘very much’)
	12. I feel tensed	-point scale (1 ‘not at all’ to 7 ‘very much’)
Self-esteem	13. I like myself	7-point scale (1 ‘not at all’ to 7 ‘very much’)
	14. I am ashamed of myself	7-point scale (1 ‘not at all’ to 7 ‘very much’)
	15. I doubt myself	7-point scale (1 ‘not at all’ to 7 ‘very much’)
	16. I am satisfied with myself	7-point scale (1 ‘not at all’ to 7 ‘very much’)
Physical well-being	17. I am tired	7-point scale (1 ‘not at all’ to 7 ‘very much’)
	18. I feel well	7-point scale (1 ‘not at all’ to 7 ‘very much’)
	19. I am in pain	7-point scale (1 ‘not at all’ to 7 ‘very much’)
	20. I have problems in walking	7-point scale (1 ‘not at all’ to 7 ‘very much’)
Activity	21. What am I doing? (just before the alert)	Doing nothing; resting; work; household; self care; caring for partner; active relaxation; passive relaxation; something else
	22. And also?	Doing nothing; resting; work; household; self care; caring for partner; active relaxation; passive relaxation; something else
	23. And…?	Doing nothing; resting; work; household; self care; caring for partner; active relaxation; passive relaxation; something else
	24. I like doing this	7-point scale (1 ‘not at all’ to 7 ‘very much’)
	25. I would rather be doing something else	7-point scale (1 ‘not at all’ to 7 ‘very much’)
	26. This is difficult for me	7-point scale (1 ‘not at all’ to 7 ‘very much’)
	27. I feel I am being active	7-point scale (1 ‘not at all’ to 7 ‘very much’)
	28. I can do this well	7-point scale (1 ‘not at all’ to 7 ‘very much’)
	29. I am doing this activity together with my partner	Yes; no
Location	30. Where am I?	At home; at family’s/friend’s place; at work; health care setting; public place; transport; somewhere else
Social company	31. Who am I with?	Partner; family; friends; colleagues; health care professional; acquaintances; strangers/others; nobody
	32. With whom else?	Partner; family; friends; colleagues; health care professional; acquaintances; strangers/others; nobody
	33. And…?	Partner; family; friends; colleagues; health care professional; acquaintances; strangers/others; nobody
	*Branching questions in case of being in company:*	
	34. I would prefer to be alone	7-point scale (1 ‘not at all’ to 7 ‘very much’)
	35. I think my company is pleasant	7-point scale (1 ‘not at all’ to 7 ‘very much’)
	36. I feel at ease in this company	7-point scale (1 ‘not at all’ to 7 ‘very much’)
	*Branching questions in case of being alone:*	
	34. I would prefer to be in company of others	7-point scale (1 ‘not at all’ to 7 ‘very much’)
	35. I enjoy being alone	7-point scale (1 ‘not at all’ to 7 ‘very much’)
	36. I feel at ease being alone	7-point scale (1 ‘not at all’ to 7 ‘very much’)
Events	37. Since the last alert the most important thing that happened is…	(take an event in mind before you continue)
	38. How pleasant was this event?	bipolar scale (−3 ‘very unpleasant’ to +3 ‘very pleasant’)
	39. I had this situation under control	7-point scale (1 ‘not at all’ to 7 ‘very much’)
	40. Was this situation unexpected?	7-point scale (1 ‘not at all’ to 7 ‘very much’)
	41. The event was important to me	bipolar scale (−3 ‘very unimportant’ to +3 ‘very important’)
	42. With whom was I?	Partner; nobody; someone else
General	43. This alert disturbed me	7-point scale (1 ‘not at all’ to 7 ‘very much’)
Morning questionnaire		
	1. I slept well	7-point scale (1 ‘not at all’ to 7 ‘very much’)
	2. How long did it take before I fell asleep	0–5 min; 5–15 min; 30–45 min; 45–60 min; 1-2 h; 2 4 h; >4 h
	3. How often did I wake up last night	1 time; 2 times; 3 times; 4 times; 5 times; more than 5 times
	4. My partner disturbed my sleep	7-point scale (1 ‘not at all’ to 7 ‘very much’)
	5. I feel rested	7-point scale (1 ‘not at all’ to 7 ‘very much’)
	6. I feel apprehensive about today	7-point scale (1 ‘not at all’ to 7 ‘very much’)
Evening questionnaire		
	1. This was an ordinary day	7-point scale (1 ‘not at all’ to 7 ‘very much’)
	2. If I had not had the device, I would have done different things today	7-point scale (1 ‘not at all’ to 7 ‘very much’)
	3. I generally felt well today	7-point scale (1 ‘not at all’ to 7 ‘very much’)
	4. I generally felt tired today	7-point scale (1 ‘not at all’ to 7 ‘very much’)
	5. I generally felt tensed today	7-point scale (1 ‘not at all’ to 7 ‘very much’)
	6. I generally worried a lot today	7-point scale (1 ‘not at all’ to 7 ‘very much’)
	7. I generally felt able to manage today	7-point scale (1 ‘not at all’ to 7 ‘very much’)
	8. My health state was good today	Visual Analogue Scale (0 ‘worst imaginable health’ to 100 ‘best imaginable health’)
	9. How many hours did you spend on caring for your partner today (incl. supervision)	0 h; 1 h; 2 h; 3 h; 4 h; 5 h; >5 h
	10. Today I felt strained in the interactions with my partner	7-point scale (1 ‘not at all’ to 7 ‘very much’)
	11. Today I felt stressed due to my care responsibilities	7-point scale (1 ‘not at all’ to 7 ‘very much’)
	12. Today I felt that the situation with my partner did not allow me as much privacy as I would have liked	7-point scale (1 ‘not at all’ to 7 ‘very much’)
	13. Today I had enough time for myself	7-point scale (1 ‘not at all’ to 7 ‘very much’)
	14. Today I was in need of support	7-point scale (1 ‘not at all’ to 7 ‘very much’)
	15. Today I received enough support	7-point scale (1 ‘not at all’ to 7 ‘very much’)
	*Today, to what extent did your partner suffer from:*	
	16. Being sad or depressed	7-point scale (1 ‘not at all’ to 7 ‘very much’)
	17. Being anxious our nervous	7-point scale (1 ‘not at all’ to 7 ‘very much’)
	18. Acting impulsively or embarrassing	7-point scale (1 ‘not at all’ to 7 ‘very much’)
	19. A loss of interest in activities/other people	7-point scale (1 ‘not at all’ to 7 ‘very much’)
	20. Being irritated or impatient	7-point scale (1 ‘not at all’ to 7 ‘very much’)
	21. Being too cheerful for no reason	7-point scale (1 ‘not at all’ to 7 ‘very much’)
	22. Being restless	7-point scale (1 ‘not at all’ to 7 ‘very much’)
	23. Agitation/aggression	7-point scale (1 ‘not at all’ to 7 ‘very much’)
	24. Beliefs that you know are not true	7-point scale (1 ‘not at all’ to 7 ‘very much’)
	25. Seeing false visions or hearing false voices	7-point scale (1 ‘not at all’ to 7 ‘very much’)

## Additional files

Additional file 1: Recommended items to address in a clinical trial protocol and related documents. (PDF 106 kb)

## Abbreviations

AE: adverse event
ANCOVA: analysis of covariance
CDR: Clinical Dementia Rating scale
CEA: cost-effectiveness analysis
CES-D: Center for Epidemiological Studies Depression Scale
Deal-id: dealing with daily challenges in dementia
EQ-5D: EuroQol-5D
ESM: experience sampling methodology
HADS: Hospital Anxiety and Depression Scale
HIV: Human Immunodeficiency Virus
ICER: incremental cost effectiveness ratios
LMM: linear mixed model
MUMC+: Maastricht University Medical Center+
NEO-FFI: NEO Five-Factor Inventory
NPI: Neuropsychiatric Inventory
NPI-Q: Neuropsychiatric Inventory Questionnaire (NPI-Q)
PDA: personal digital assistant
PMS: Pearlin Mastery Scale
PSS: Perceived Stress Scale
PwD: people/persons with dementia
QALY: quality-adjusted life-year
RCT: randomized controlled trial
RUD-lite: Resource Utilization in Dementia – shortened version
SAE: serious adverse event
SCQ: Sense of Competence Questionnaire
SSCQ: Short Sense of Competence Questionnaire
UCL: Utrechtse Coping List
